# Current management and prognostic factors in physiotherapy practice for patients with shoulder pain: design of a prospective cohort study

**DOI:** 10.1186/1471-2474-14-62

**Published:** 2013-02-11

**Authors:** Yasmaine H J M Karel, Wendy G M Scholten-Peeters, Marloes Thoomes-de Graaf, Edwin Duijn, Ramon P G Ottenheijm, Maaike P J van den Borne, Bart W Koes, Arianne P Verhagen, Geert-Jan Dinant, Eric Tetteroo, Annechien Beumer, Joost B van Broekhoven, Marcel Heijmans

**Affiliations:** 1 Faculty of Health, Research group Diagnostics, Avans University of Applied Sciences, PO Box 90.1164800, RA, Breda, The Netherlands; 2 Department of General Practice, Erasmus Medical Center, Rotterdam, The Netherlands; 3 Department of General Practice, CAPHRI School for Public Health and Primary Care, Maastricht University, Maastricht, The Netherlands; 4 Department of Orthopaedic Surgery, Amphia Hospital, Breda, The Netherlands; 5 Department of Radiology, Amphia Hospital, Breda, The Netherlands; 6 Fysiocollectief, Prinsenbeek, The Netherlands; 7 Fysiomaatwerk, Heeswijk-Dinther, The Netherlands

## Abstract

**Background:**

Shoulder pain is disabling and has a considerable socio-economic impact. Over 50% of patients presenting in primary care still have symptoms after 6 months; moreover, prognostic factors such as pain intensity, age, disability level and duration of complaints are associated with poor outcome. Most shoulder complaints in this group are categorized as non-specific. Musculoskeletal ultrasound might be a useful imaging method to detect subgroups of patients with subacromial disorders.

This article describes the design of a prospective cohort study evaluating the influence of known prognostic and possible prognostic factors, such as findings from musculoskeletal ultrasound outcome and working alliance, on the recovery of shoulder pain. Also, to assess the usual physiotherapy care for shoulder pain and examine the inter-rater reliability of musculoskeletal ultrasound between radiologists and physiotherapists for patients with shoulder pain.

**Methods:**

A prospective cohort study including an inter-rater reliability study. Patients presenting in primary care physiotherapy practice with shoulder pain are enrolled. At baseline validated questionnaires are used to measure patient characteristics, disease-specific characteristics and social factors. Physical examination is performed according to the expertise of the physiotherapists. Follow-up measurements will be performed 6, 12 and 26 weeks after inclusion. Primary outcome measure is perceived recovery, measured on a 7-point Likert scale. Logistic regression analysis will be used to evaluate the association between prognostic factors and recovery.

**Discussion:**

The ShoCoDiP (Shoulder Complaints and using Diagnostic ultrasound in Physiotherapy practice) cohort study will provide information on current management of patients with shoulder pain in primary care, provide data to develop a prediction model for shoulder pain in primary care and to evaluate whether musculoskeletal ultrasound can improve prognosis.

## Background

This paper describes the ShoCoDiP (Shoulder Complaints and the use of Diagnostic Ultrasound in Physiotherapy practice) cohort study. Publishing the design of a study provides insight into the objectives and procedures before publishing the results. It may also protect against (subconscious) selective outcome reporting. Shoulder disorders are the second most common musculoskeletal complaint in the general population with a point prevalence of 20.6% [1] and cause considerable functional disability, pain and healthcare costs [2]. The reported 12-month prevalence for shoulder disorders is 6.7 to 66.7% [3]. In the Netherlands, the annual incidence in general practice is 15-16/1000 person-years [4]. About 30-40% of the patients with shoulder pain consult a general practitioner (GP) due to these complaints [1]. Chronicity and recurrence of shoulder pain are common [5-7]. About 40% of the patients still experience pain after 12 months [6] and 40% re-consult their GP [2]. There is strong evidence that prognostic factors for shoulder pain such as age, high disability scores, duration of shoulder pain and pain intensity are associated with poor outcome [4,8,9]. Having a specific diagnosis like bursitis, rotator cuff tear and frozen shoulder is reported to be a predictor for increased recovery in patients with upper extremity disorders compared to patients with a non-specific diagnosis in general practice [8].

At first consultation GPs recommend a ‘wait and see’ policy in about 40% of the patients, 39% receive oral NSAIDs and 16% are referred for physiotherapy [10]. Early treatment in general practice mainly consists of pain medication and advice [2]. The guideline for shoulder pain of the Dutch College of General Practitioners advises a referral for physiotherapy or a corticosteroid injection as a standard procedure in shoulder pain when these complaints are present for ≥ 2 weeks [2]. In the Netherlands, since 2006 patients can directly access physiotherapy care which means they do not need a referral to consult a physiotherapist (PT). Nevertheless, the Dutch institute for paramedical care reported that in 2009 49% of the patients who visited the PT were referred by their GP, 38% used self-referral, and the remaining 13% were referred by a medical specialist [11].

In primary care, the information gained during history taking and physical examination is used to make a diagnosis and decide on treatment options. Unfortunately, physical examination is not always a reliable or valid diagnostic tool [12-14]. As a result, most complaints are regarded as non-specific, because no specific pathology can be diagnosed. When additional diagnostic information is needed, GPs can refer patients to radiologists for further diagnostic imaging, such as musculoskeletal ultrasound (MSU).

Nowadays, in the Netherlands many PTs attend additional courses on MSU, which can be a reliable and relatively inexpensive tool for the diagnosis of patients with shoulder pain [15,16]. A recent systematic review shows that MSU has a sensitivity of 95% and a specificity of 96% for full thickness rotator cuff tears, and a sensitivity of 72% and specificity of 93% for partial thickness tears when performed by an experienced radiologist [17]. Therefore, MSU performed by an experienced examinator might help in accurately diagnosing rotator cuff tears. Knowing this, the question remains whether or not patients will respond better to treatment once this pathology is identified in primary care. An accurate diagnosis is essential to ensure that patients receive appropriate treatment and correct information about their prognosis. Apart from the proposed treatment, the prognosis can be influenced by the patient’s experience in the perceived health care or acquired treatment goals. This involves a therapeutic encounter between the patient and PT, hereafter referred to as ‘working alliance’. A recent systematic review indicated that working alliance has a consistent positive correlation with treatment outcome in a physical rehabilitation setting [18]. The present study will evaluate whether working alliance and pathology detected on MSU are possible prognostic factors in primary care patients with shoulder pain.

MSU used by PTs probably could help to identify subgroup of patients who might better respond to physiotherapy treatment. We assume that a more specific diagnosis will lead to more specific treatment choices and better patient prognosis. Classifying these shoulder disorders seems to be a diagnostic challenge and therefore a shift from diagnostic research to prognostic research might help in the first steps of consultation [19].

The primary aim of the ShoCoDiP study is to evaluate physiotherapy care and prognostic factors in patients with shoulder pain and investigate whether MSU and the working alliance are related to patient recovery. Secondary aims are to assess the inter-rater reliability of MSU between PTs and radiologists, and whether patient characteristics of those who receive MSU differ from those who do not receive MSU.

## Methods

### Design

**Figure 1:**
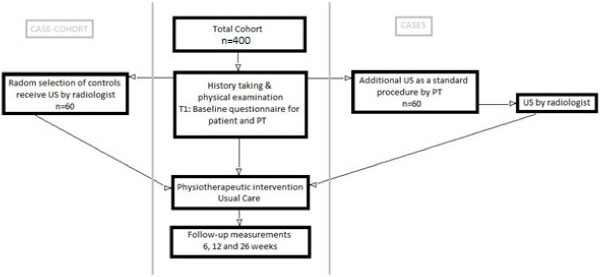
Flow chart of the study protocol.

A prospective cohort study including patients with shoulder pain presenting in primary care physiotherapy (Figure 1). Furthermore, a nested case cohort design will be used to evaluate whether patient characteristics differ between patients who do and do not receive MSU (Figure 1). The control group will be randomly selected from the total cohort. These patients are matched to patients who received MSU, based on the PT’s decision, by age and sex. Patients who received MSU via the PT are also scanned by a radiologist to evaluate the inter-rater reliability. The Medical Ethics Committee of the Erasmus Medical Center in Rotterdam approved the study protocol (MEC-2011-414).

### Recruitment of PT, radiologists and patients

#### Physiotherapists (PTs)

PTs in the southwest region of the Netherlands will be asked to participate in the study. An introductory meeting was organized to explain study procedures and data collection. Selection criteria for PTs using MSU are: 1)PTs having ≥ 1 year of experience after their MSU course, 2) PTs performed ≥ 100 MSU examinations of the shoulder, 3) the transducer should have a minimum frequency of 7.5 MHz, and 4) having appropriate software (beamforming technology). These PTs were trained to use the MSU protocol by Jacobson [20] during a special consensus meeting.

#### Radiologists

Radiologists in the southwest region of the Netherlands are invited by telephone and email. Only radiologists who are specialized in musculoskeletal radiology and perform MSU in their hospitals are invited to participate. A total of 9 radiologists from 4 hospitals participate in the study. One of the researchers visits to inform them about the study procedures and the MSU protocol as described by Jacobson [20].

#### Patients

From November 2011 to November 2012 PTs will recruit consecutive patients in primary care. Patients eligible for the study suffer from shoulder pain, are aged ≥ 18 years and have adequate understanding of the Dutch language. Patients are excluded if they have serious pathologies (infection, cancer or fracture), previous surgery of the shoulder in the last 12 months, or received diagnostic imaging techniques such as MSU, MRI or X-ray of the shoulder in the 3 months prior to start of the study. All patients provided written informed consent.

### Data collection

Data will be collected using online Limeservice software and safely stored by both the investigators and the software holders. Patients will receive a digital questionnaire at baseline and at 6, 12 and 26 weeks after inclusion. PTs receive questionnaires at baseline and at 3, 6 and 12 weeks follow-up. Whenever a PT performs a MSU, within 1 week the patient will undergo a second MSU by a blinded radiologist. To reduce the chance of missing data, an email reminder will be sent at 2 and 5 days to the patient or PT. Newsletters will be sent every month to the participating PTs to encourage adherance to the study. Moreover, all PTs will be contacted by telephone every 3 months to ensure adherence to the study protocol, and stimulate them to recruit eligible patients.

### Baseline assessment

Patient characteristics, prognostic factors and disease-specific information will be collected at baseline. These include demographic variables and complaint-specific variables. PTs will report data on physical examination and their interpretation after history taking and physical examination. Possible hypotheses are described in Table 1.

**Table 1:** Hypotheses

0	Possible sub-acromial impingement
1	Possible internal (posterior) impingement
2	Possible instability of the glenohumeral joint
3	Possible SLAP lesion
4	Possible biceps tendinopathy
5	Possible frozen shoulder/capsulitis
6	Possible disorder of cervic-thoracic spinal column and adhering costae
7	Possible myofascial trigger point in neck and shoulder region
8	Possible disorder of the acromioclavicular/sternoclavicular joint
9	Possible hypertonia in neck/shoulder region
10	Possible strain or sprain in neck/shoulder region
11	Not possible to specify a clear hypothesis
12	Other non-specified

Hypotheses are built and edited based on the clinical opinions of 5 physiotherapists.

### Prognostic factors

Possible prognostic factors on recovery for patients with shoulder pain are extracted from the literature [4,21-24] and consist of pain intensity, duration of complaints, age, gender, disability, highest level of education, job description (physically heavy work, static repetitive work or work with awkward postures; yes/no), sick leave due to shoulder complaint (yes/no), and complaints worsen during work (yes/no). Also, exploratory MSU outcome and the Dutch version of the working alliance inventory (WAV-12) will be assessed as possible prognostic factors as they might be related to patient recovery.

### Physiotherapy management

Descriptive factors like the frequency of diagnostic hypotheses, the treatment period, costs, and treatment goals and related interventions in physiotherapy practice will be assessed in the PT questionnaire.

### Sample size

Based on the literature about 40% of the patients with shoulder pain will recover within 6 months [4]. We will estimate to include 15 prognostic variables in our prognostic model. Based on the 1 in 10 rule of 10 events per variable, a total of 150 events are needed in the smallest outcome (recovered or not). Therefore, the total study population should include about 300 subjects. Adjusting for about 20% missing values, the total population will comprise a minimum of 400 subjects.

### Outcome measures

#### Primary outcome

Our primary outcome is recovery measured with the Global Perceived Effect (GPE) scale [25] (Table 2). The GPE uses a 7-point Likert scale scoring whether the patient’s condition has improved or deteriorated since the start of their physiotherapy treatment. This scale ranges from ‘worse than ever’ to ‘fully recovered’. Patients are considered to be recovered when they score ‘strongly improved’ or ‘completely recovered’ [25].

**Table 2:** Baseline to follow-up measures

	***Baseline***	**T1: *3 weeks***	***T2: 6 weeks***	***T3: 12 weeks***	***T4: 6 months***
Inclusion/exclusion criteria	**X**				
Demographic data	**X**				
GPE		**X**	**X**	**X**	**X**
SPS	**X**	**X**	**X**	**X**	**X**
SDQ-NL	**X**	**X**	**X**	**X**	**X**
SPADI	**X**	**X**	**X**	**X**	**X**
EQ5D	**X**	**X**	**X**	**X**	**X**
WAV-12			**X**		
Medical consumption	**X**		**X**	**X**	**X**
**Physiotherapist**					
Interpretation from physical examination and patient history	**X**				
Change in treatment plan	**X**	**X**	**X**	**X**	
Treatment goals	**X**	**X**	**X**	**X**	
Number of treatments	**X**	**X**	**X**	**X**	

*GPE*: General Perceived Effect, *SPS*: Shoulder Pain Score, *SDQ-NL*: Dutch Shoulder Disability Questionnaire, *SPADI*: Shoulder Pain and Disability Index, *EQ5D*: Euroquol five-item quality of life questionnaire, *WAV-12*: Dutch Working Alliance Scale (Short Form).

#### Secondary outcome

Functional disability will be measured with the Dutch version of the Shoulder Disability Questionnaire (SDQ-NL). The SDQ has 16 items which are answered with either ‘yes’, ‘no’, or ‘not applicable’. The score ranges from 0 to 100, with a high score indicating more functional disability. This questionnaire has good construct validity [23], and appears to be a useful discriminative instrument in primary care [26]. The Shoulder Pain Disability Index (SPADI) is measured in conjunction with the SDQ-NL to validate the SPADI questionnaire in Dutch. The SPADI has 8 questions designed to measure the degree of difficulty someone has with various activities of daily living that require the use of upper extremities. Internal consistency is good (Cronbach’s alpha: 0.90). Test-retest reliability of the SPADI and the intraclass correlation for the disability subscale ranges from 0.57-0.84 [27].

Pain severity will be assessed with the Shoulder Pain Score (SPS); this instrument has 6 questions about pain symptoms experienced in the last 24 hours scored on a 4-point scale, and an 11-point Numeric Rating Scale. Internal consistency for the SPS is good (Cronbach’s alpha: 0.82) [28].

Health-related quality of life will be measured using the Euroquol five-item quality of life questionnaire (EQ-5D) [29]. This questionnaire covers 5 dimensions of health, and a visual analogue scale ranging from 0–100. The five dimensions of health are: mobility, self-care, usual activities, complaints/discomfort and anxiety/depression. The patient can score three levels of severity in each dimension (1 = no problem, 2 = moderate problem, 3 = severe problem). Scoring will be calculated according to the European guideline recommendations [30].

Working alliance will be measured with a Dutch version of the Working Alliance Inventory (WAV-12). The WAV-12 will be assessed after 6 weeks. This questionnaire has three subscales designed to assess three primary components of the working alliance: 1) how closely client and therapist agree on and are mutually engaged in the goals of treatment (Cronbach’s alpha: 0.85), 2) how closely client and therapist agree on how to reach the treatment goals (Cronbach’s alpha: 0.83), and 3) the degree of mutual trust, acceptance, and confidence between client and therapist. Patients score on a 5-point scale ranging from rarely to always [31,32].

MSU will be standardized in terms of 11 primary outcome categories: 1) tendinopathy, 2) calcification, 3) full or 4) partial thickness tear, 5) Biceps tendon tear, 6) subacromial-subdeltoid bursitis, 7) subacromial impingement, 8) osteoarthritis of the acriomio-clavicular joint, 9) cortical discontinuity of superior aspect of the acromion, 10) no specific pathology, or 11) other. In case a diagnosis in category 1–2 was made, it could be specified in the following diagnostic subgroups; supraspinatus, infraspinatus, teres minor or subscapularis and biceps tendon. For category 3–4 it could be specified in; supraspinatus, infraspinatus and teres minor or subscapularis tendon. This resulted in a total of 11 diagnostic categories (Table 3) [17].

**Table 3:** Musculoskeletal ultrasound imaging outcomes

**Pathology**	**Anatomical site**
1. Tendinopathy	supraspinatus tendon
	subscapularis tendon
	infraspinatus tendon
	teres minor tendon
	long head biceps tendon
2. Calcification	supraspinatus tendon
	subscapularis tendon
	infraspinatus tendon
	teres minor tendon
	long head biceps tendon
3. Full-thickness tear	supraspinatus tendon
	subscapularis tendon
	infraspinatus tendon
	teres minor tendon
4. Partial-thickness tear	supraspinatus tendon
	subscapularis tendon
	infraspinatus tendon
	teres minor tendon
5. Biceps tendon tear	
6. Subacromial-subdeltoid bursitis (>2 mm low frequency)	
7. Subacromial impingement (upon active abduction)	
8. Osteoarthritis	acriomio-clavicular joint
9. Cortical discontinuity	superior aspect of the acromion
10. No specific pathology	
11. Other non-specified	

### Statistical analysis

Descriptive statistics, including frequencies for categorical variables and means with standard deviations (SD) for continuous variables, will be used to describe the characteristics of the patients, PTs and radiologists. We intend to develop a prognostic model using logistic regression analysis with recovery (GPE) after 6 months as the primary outcome. Missing values will be handled using multiple imputation techniques. All candidate predictors will be included in our prognostic model. All assumptions (homogeneity of variance, independence-normality of residuals, linearity and multicollinearity) for building a regression model will be checked before model building. Internal validation of the model will be assessed by a bootstrap procedure (200 repetitions) to assess the accuracy of the regression analysis. The inter-rater reliability will be evaluated with a KAPPA statistic. Statistical analysis will be performed using SPSS 20.0. A p-value of >0.05will be considered statistically significant.

## Discussion

The proposed study will describe the current management of shoulder pain in primary care and will help to determine which factors can predict patient recovery in PT practice. This study is designed to include key methodological features in order to minimize bias. These features include sampling of a representative cohort from physiotherapy setting with a high rate of follow-up.

Based on the sample of patients that will be recruited from physiotherapy practices, we aim to produce a pragmatic prediction model for PTs in primary care.

Possible prognostic factors and confounders are selected based on previous research [4]. The selected population of PTs in primary care enables us to include possible additional predictors such as characteristics from the PT and ultrasonographer. All medical consumption besides physiotherapy will be registered during follow-up questionnaires. Completeness of data collection will be stimulated by means of email reminders.

Although we will select a heterogeneous group of patients with shoulder complaints, we stress two important exclusion criteria. The first is that patients who had surgery of the shoulder in the previous 12 months are excluded, since these patients seem to differ in pathology and prognosis. Excluding these patients will ensure a more valid prediction model. Secondly, we postulate that PTs base their diagnosis and interventions on imaging techniques that were performed in the past; moreover, in case of the inter-rater reliability study, this could threaten blinding because most patients know the results of diagnostic imaging. Therefore, this study also excludes patients who had imaging of the shoulder in the 3 months prior to the start of physiotherapy treatment. PTs will be instructed to act as usual and are not instructed to adhere to a specific intervention protocol. This study aims to report on usual care in physiotherapy practice and provide insight into the diagnostic and therapeutic management of patients. Because patients are selected in primary care physiotherapy, we assume that they will represent the usual population consulting the PT with shoulder pain.

Patients in the control group will be randomly matched (by age and sex) to patients that receive an MSU by their PT. To avoid disease progression bias, their second MSU will be performed within 1 week; we do not expect that partial or full-thickness ruptures or calcifications will heal within 1 week. However, we cannot be certain that patient recovery is related to changes in patho-anatomical findings on MSU. Furthermore, the literature describes a high prevalence of rotator cuff tears in asymptomatic populations [33,34]. Therefore, we cannot ensure that these pathologies found on MSU images cause symptoms or constraints in daily activities for patients.

Radiologists and PTs will be blinded to each other’s findings. Moreover, they will be blinded to clinical information that was not intended to form part of the MSU assessment. Radiologists are instructed to keep the patient blinded from MSU outcome. Blinding will be evaluated in the follow-up questionnaire of the patient.

From previous research it is known that MSU is operator dependent [35]. PTs and radiologists are instructed to use a standardized scanning protocol [20], to ensure comparability in MSU procedures. Current management with MSU does not standardize pathology criteria. To assess the effect of current management of MSU in primary care we chose not to define criteria for pathology in this study. Nevertheless, we standardized possible outcome definitions for both the radiologist and PT in order to be able to categorize data.

We assume that inter-rater reliability between PT and radiologist might be influenced by the quality of ultrasound equipment and experience. Therefore, only equipment with transducer frequencies of at least 7.5 MHz will be used in physiotherapy practice and PTs should have at least 1 year of experience with ≥ 100 examinations of the shoulder.

Until now, reliability studies generally evaluated the inter-rater reliability between radiologists. However, PTs increasingly use MSU in daily practice and the reliability between different professions has not yet been evaluated.

It is hoped that this prospective cohort study will help improve the current management and prognosis of patients with shoulder pain.

## Abbreviations

ShoCoDiP: Shoulder Complaints and Diagnostic Ultrasound In Physiotherapy; GP: General practitioner; PT: Physiotherapist; MSU: Musculoskeletal ultrasound imaging; MHz: Mega hertz; WAV-12: Dutch working alliance inventory; GPE: Global Perceived Effect; SDQ-NL: Dutch Shoulder Disability Questionnaire; SPADI: Shoulder Pain And Disability Questionnaire; SPS: Shoulder Pain Score; EQ-5D: Euroqol five-item quality of life questionnaire.

## Competing interests

The authors declare that they have no competing interests.

## Authors’ contributions

*YK* will collect the data together with MT and ED. YK will perform the analyses and wrote the manuscript together with WS and AV. YK and MT have full access to all the study data. *WS* is the principal investigator of the study and contributed to the design and coordination of the study and will contribute to the interpretation of the study results. *MT* collected part of the data, will perform part of the analyses and interpretation of the results. *ED* collected part of the data, will perform part of the analyses and interpretation of the results. *RO* is an expert in the field of musculoskeletal complaints and MSU, contributed to writing the manuscript and will contribute to the interpretation of the results. *BK* is an expert in the field of epidemiology and contributed to the design and will contribute to the interpretation of the results. *AV* is principal investigator and an expert in the field of epidemiology and contributed to the design and coordination of the study and will contribute to the interpretation of results. All other members from the ShoCoDiP study group *GD*, *AB*, *MB*, *ET*, *JB and MH* reviewed the manuscript, but did not meet the criteria for authorship. All authors read and approved the final manuscript.

## Pre-publication history

The pre-publication history for this paper can be accessed here:

http://www.biomedcentral.com/1471-2474/14/62/prepub
